# A case of aberrant bilateral vertebral arteries orgin presenting with right VA dissection

**DOI:** 10.22088/cjim.11.1.116

**Published:** 2020

**Authors:** Maryam Bahadori, Seyedeh Fahimeh Shojaei, Rezan Ashayeri, Sara Esmaeili, Masoud Mehrpour

**Affiliations:** 1 Department of Neurology, Firoozgar Hospital, Iran University of Medical Sciences, Tehran, Iran; 2 Firoozgar Clinical Research Development Center (FCRDC), Firoozgar Hospital, Iran University of Medical Sciences, Tehran, Iran

**Keywords:** Aberrant origin, Vertebral artery variation, Embryology

## Abstract

**Background:**

Knowledge of variations in the origin of vertebral artery (VA) is indispensable to vascular surgeons. Aberrant origin of vertebral artery on either side is an uncommon finding. There are unilateral and bilateral variability in VA origin.

**Case presentation:**

We present a case of vertebral artery dissection who was found to have bilateral VAs aberrant origin. The right VA took origin from the right common carotid artery (CCA) which is a completely a rare finding, and the left VA originated from the arch of aorta.

**Conclusion:**

*Unlike most similar reported cases, the VA diameter at origin was larger on the left than on the right side. The possible embryological mechanism is discussed.*

The vertebral arteries (VA) are important arteries of the neck, which typically originate from the posterosuperior aspect of the first part of the subclavian artery. Each vessel enters the foramen transversium of C6 and inside the skull they merge to form the basilar artery (1). There are variations in the course, size, and origin of the VA. This artery is a common site for vascular surgeries or diagnostic procedures in head and neck region. Hence, it is crucial to be aware of such possible origins and courses of VAs when they are not found in their normal anatomic place. Herein we present a case of right VA dissection in a 39-year-old woman whose catheter angiography revealed an anomalous origin of both VAs. Left VA originated from the aortic arch and the right one originated from the right common carotid artery (CCA). Aortic arch as the origin of left VA is a well-known variation, but very few cases of aberrant right VA from right CCA have been published to date, and the coincidence of these two are even much rarer.

## Case Presentation

A 39-year-old woman was referred to Firoozgar General Hospital15 days after having a head trauma in a car accident. Her initial symptom was occasional headaches which within 36 hours were followed by a thunderclap headache along with left side hemiparesis, diplopia and lethargy. The brain computed tomography (CT) scan at previous center revealed hypodensity in pons and cerebellum. Her history and medical findings were highly suggestive of right vertebral artery dissection, and she was actually referred to our center as a hospital due to the availability of cerebral artery angiography. On admission day in our center, she was lethargic and disoriented and her GCS was 9. Neurologic examination revealed conjugate gaze palsy to the left and impaired adduction in the left eye. Vertical eye movements were normal. Her muscle forces were 2/5 and 3/5 in the left upper and lower extremities, respectively. Her NIHSS score (National Institutes of Health Stroke Scale) was 22 at the time of admission.

**Figure 1:**
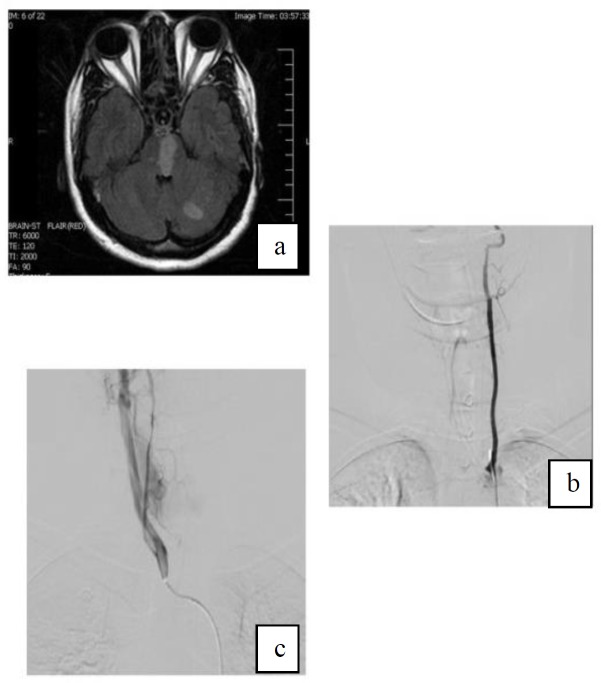
a) Axial T2-FLAIR weighted MRI depicts hypersignality in left cerebellum and medulla b) Cerebral DSA shows aberrant origin of left VA from aortic arch between left subclavian artery and left CCA c) Cerebral DSA realizes dissection in V2 and tapering at V4 due to occlusion of the lumen

Magnetic resonance imaging (MRI) of the brain showed hypersignality in paramedian of pons, vermis, right and left hemispheres of the cerebellum (figure 1a). She went on aspirin 80 mg daily, clopidogrel 75 mg twice daily and unfractionated heparin (UFH) 5000 units twice daily. Transcranial duplex sonography showed absent flow in right VA with normal flow velocity and direction in left VA, basilar artery, and bilateral carotid arteries.

Cerebral catheter angiography was done for her on the third day of admission. The left VA originated from aortic arch between the left subclavian artery and left CCA (figure 1b). Left VA prevertebral length was 35.6 mm and its diameter at origin was 5.5 mm. The right vertebral artery was seen arising from the right common carotid artery, a rare variation, and there was dissection in V2 part and sudden tapering at V4 part because of occlusion of the lumen (figure 1c). The length of the prevertebral part of right VA was 19.9 mm, and its diameter at the origin was 3.6 mm.

Other arteries including CCAs, internal and external carotid arteries, middle cerebral arteries, anterior cerebral arteries and posterior cerebral arteries were normal. After angiography, asprin and clopidogrel were discontinued UFH 1000 units per hour were infused intravenously. Control CT after angiography revealed no intracerebral hemorrhage (ICH). The patient was discharged after 26 days with rivaroxaban 20 mg/daily, captopril 50 mg/daily, metoprolol 12.5 mg daily and atorvastatin 40 mg/daily. At discharge time, she was awake and alert, her gaze palsy and limb weakness improved but not completely resolved and NIHSS was 12. On the 9th month of follow up, left extremity forces were 4/5 and she was still complaining about diplopia. Her NIHSS was evaluated as 5.Modified Rankin Scale (mRS) at the time of discharge was 2 and after 9 months it was one.

## Discussion

**Figure 2:**
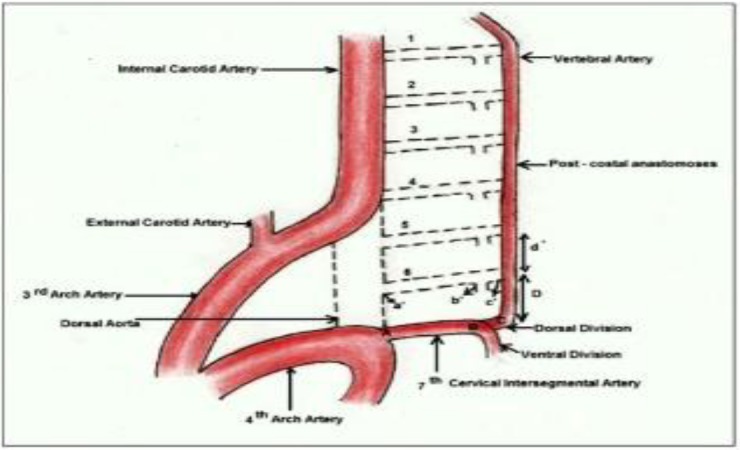
Schematic diagram of embryological development of Vertebral Artery origin from Subclavian Artery

Both vertebral arteries usually arise from subclavian arteries, but there are reports of different variations in VA origin. The origin of the left VA from the aortic arch is a well-defined variation, and the prevalence of this variation has been reported in a very vast range in different studies. In a recent autopsy-based study it was found in 14.8% of 27 cases with the female predominance (2). However, anomalous origin of the right VA is very rare; it can arise from aorta, carotid arteries, brachiocephalic artery or those originating from duplicated origin (3). The prevalence of right CCA as the origin of right VA has been reported 0.18% (4). To understand these anomalies, embryological mechanisms of vascular development should be discussed. When the embryo is 7mm, development of vertebral arteries begins, and 7 cervical intersegmental arteries (CIA) jump out of dorsal aorta. The vertebral artery is formed by the longitudinal anastomosis that links these arteries and starts elongating toward the base of the brain. The 6 CIAs obliterate but the seventh arteries remain and become the subclavian arteries. This embryological process results in the rising of VA from subclavian artery (5) (figure 2).

His developmental process variation leads to different vertebral artery origins. If the first or the second CIA fails to involute, the vertebral artery will originate from the internal or external carotid artery. If the third through the sixth CIAs persist, the result is anomalous origin from the common carotid artery or aortic arch (6). In our case, the left sixth CIA may not obliterated, probably due to the failure of developing an anastomose between the 6th and the 7th CIAs (1, 2). As a result, this patient's left VA began from the aortic arch. Also, on the right side, the sixth CIA persistence resulted in the aberrant origin of right VA from right CCA (4). So, it seems that in this patient bilateral sixth CIAs did not regress during the embryonic period.

In a recent review article, 66 different patterns were found for variable origins of VAs among which 17.54% were related to cases with aberrant origins of both right and left VAs. In this rare group, the most prevalent cases were those similar to our case whose aortic arch was the origin of left VA, and right CCA as the origin of right VA (figure 5). To our knowledge, 15 similar cases have been reported before our case, and it is still considered a very rare finding. These similar cases had different initial symptoms including: headache, vertigo, personality change, stroke, hemisensory disturbance or numbness, chest pain, swallowing difficulties. One patient was also found during aortic valve replacement (7). In some studies, it has been discussed that aberrant vertebral arteries may become more prone to dissection and this issue was referred to hemodynamic alteration and tolerating more shear stress on the vessel wall due to longer course of the vessel (10, 11). 

In a review article, it was mentioned that VA aberrancy is not always associated with clinical symptoms. However, some studies have reported a higher risk of dissection in VA variations (7). Considering the diameter of arteries, previous studies of aberrant bilateral vertebral arteries origin reported that the right VA had a larger caliber than the left VA (7). However, in our case, the diameters of VAs at the origin were 5.5 mm and 3.6 mm in the left and the right Vas, respectively, which shows that the left VA diameter was larger than the right side, unlike some previous reports. Another point is that in most cases the diameter of VA arising from aortic arch is smaller than VAs from the normal subclavian origin. The normal diameter of VAs at origin has been reported between 5.5 and 6.9 in studies (8, 90. Whereas in our case the left VA from aortic arch had the normal diameter at the origin. Probably because of the right VA anomaly association and the smaller size of the right side, compensatory left VA became the dominant artery and ended up being larger than the right side to supply brain circulation.

In conclusion**, u**nderstanding of cerebrovascular circulation anomalies is important for both endovascular intervention and surgical planning. In addition, it is possible that patients with vertebral artery variations are more prone to dissection than normal ones.
